# Effects of probiotics, prebiotics, and synbiotics on lung function and symptoms in adults with bronchial asthma: a systematic review and meta-analysis

**DOI:** 10.3389/fimmu.2026.1896757

**Published:** 2026-08-04

**Authors:** Qianqian Zhang, Nianzhi Zhang, Ying Zheng, Jing Zhou, Ling Liu, Gang Dong

**Affiliations:** 1 The First Clinical Medical College, Anhui University of Chinese Medicine, Hefei, China; 2 Department of Respiratory Medicine, The First Affiliated Hospital of Anhui University of Chinese Medicine, Hefei, China

**Keywords:** asthma, meta-analysis, prebiotics, probiotics, synbiotics, systematic review

## Abstract

**Background:**

Probiotics, prebiotics, and synbiotics may exert beneficial effects on asthma by modulating the gut microbiota and the “gut-lung axis”. However, no systematic review or meta-analysis has specifically focused on adult asthma patients.

**Objective:**

To systematically evaluate the effects of probiotics, prebiotics, and synbiotics on lung function, symptom control, and inflammatory markers in adults with bronchial asthma.

**Methods:**

Five electronic databases were systematically searched for relevant randomized controlled trials (RCTs) up to May 2026 (PROSPERO registration: CRD420261412024). Standardized mean differences (SMDs) were pooled using a random-effects model. Outcomes for which quantitative data could not be extracted were summarized narratively. The Cochrane RoB 2.0 tool and GRADE framework were used to evaluate risk of bias and grade evidence quality for primary endpoints, respectively.

**Results:**

13 RCTs were included, 7 of which provided extractable continuous quantitative data. Pooled results demonstrated significant elevations in absolute FEV_1_ (SMD=0.71, 95%CI 0.29–1.13, P=0.020) and FEV_1%_ predicted (SMD=0.51, 95%CI 0.06–0.96, P=0.026). Subgroup analyses identified numerically larger lung function gains for multi-strain formulations and 4-week interventions; no significant between-subgroup difference was observed for intervention type (P=0.462), while a significant difference was found between 4-week and 8-week treatment durations (P=0.002). The pooled change in ACT scores was not statistically significant, and this analysis was accompanied by high inter-study heterogeneity. A reduction in FeNO was observed in only a single, small trial that combined synbiotics with allergen immunotherapy, and no consistent alterations of IL-5 and IgE were detected across included studies. No severe adverse events were reported, and GRADE evidence certainty ranged from low to moderate.

**Conclusion:**

Moderate-quality evidence supports a mild beneficial effect of multi-strain *Bifidobacterium/Lactobacillus* probiotics or synbiotics on lung function in adult asthma; however, the current evidence does not justify their routine clinical use. Large long-term RCTs are required to confirm efficacy and establish optimal regimens.

**Systematic review registration:**

https://www.crd.york.ac.uk/PROSPERO/, identifier CRD420261412024.

## 1 Introduction

Bronchial asthma is a chronic respiratory disease characterized by variable airflow limitation, airway hyperresponsiveness, and chronic airway inflammation, with recurrent wheezing, shortness of breath, chest tightness, and cough as its main clinical manifestations (1). Epidemiological data indicate a continuous rise in the global prevalence of asthma, posing a significant global public health challenge (2). Currently, first-line pharmacological treatments for asthma are mainly inhaled corticosteroids and β2-agonists, which are used to control acute symptoms and improve lung function. However, as asthma requires long-term standardized management, patients may experience adverse effects such as decreased bone density and respiratory tract infections during prolonged medication use, and may even develop drug dependence or steroid resistance (3). Therefore, exploring effective diagnostic and therapeutic strategies for asthma has become one of the critical issues urgently requiring clinical resolution.

In recent years, increasing evidence has indicated that the gut microbiota plays a central role in the development of asthma. Gut dysbiosis is not only closely related to a biased Th2 immune response but also further regulates host inflammation and immune homeostasis by influencing the production of metabolites such as short-chain fatty acids (SCFAs) (4). This immune interaction and metabolic communication mechanism between the gut and the lungs has been summarized as the “gut-lung axis” concept. This suggests that intervention strategies targeting the gut microbiota may offer a new direction for asthma prevention and treatment (5).

Probiotics, prebiotics, and synbiotics are the main means of regulating the gut microecology. They exert immunomodulatory and anti-inflammatory effects by replenishing beneficial bacteria, inhibiting pathogenic bacterial colonization, enhancing the intestinal barrier, and producing SCFAs and other metabolites (6–8). Clinical studies have shown that probiotic or prebiotic supplementation can help improve the gut microbiota of asthma patients, enhance lung function, and control inflammation (9–11). However, the results across different studies are markedly inconsistent: some randomized controlled trials (RCTs) have reported clear clinical benefits, while others have found no significant improvement in lung function or symptoms (12). Such discrepancies can be attributed to variations in bacterial strains, intervention dosages and treatment duration, alongside limited sample sizes and incomplete outcome reporting across eligible trials.

Although systematic reviews have confirmed the potential benefits of microecological preparations in pediatric asthma (13, 14), a systematic review specifically focusing on adult asthma patients and covering all three types of interventions—probiotics, prebiotics, and synbiotics—has not yet been reported. This partly reflects the fragmentation and heterogeneity of clinical evidence in the adult population. Against this background, the current systematic review and meta-analysis was performed to quantify pooled changes in lung function and asthma control among adult patients receiving microecological adjuvant treatment. Prespecified subgroup analyses were performed to explore efficacy differences across intervention types and treatment durations, aiming to generate standardized and robust evidence supporting adjuvant clinical management of adult asthma.

## 2 Methods

This systematic review followed the PRISMA 2020 guidelines (see **Supplementary Table 1**), and the study protocol was registered on PROSPERO (registration number: CRD420261412024).

### 2.1 Inclusion criteria

The PICOS (Participants, Interventions, Comparisons, Outcomes, Study design) principle was applied:

Population (P): Adults (≥18 years) with a confirmed diagnosis of asthma, irrespective of severity, duration, or asthma phenotype.

Intervention (I): Probiotics, prebiotics, or synbiotics, used alone or as an adjunct to usual care.

Comparison (C): Placebo, no intervention, or usual care (with the same background treatment in both groups).

Outcomes (O): At least one of the following: lung function parameters including forced expiratory volume in 1 second (FEV_1_), forced vital capacity (FVC), peak expiratory flow (PEF), and FEV_1_/FVC ratio; symptom/control scores such as Asthma Control Test (ACT), Asthma Control Questionnaire (ACQ), or Asthma Quality of Life Questionnaire (AQLQ); inflammatory markers including fractional exhaled nitric oxide (FeNO), interleukin-5 (IL-5), immunoglobulin E (IgE), and eosinophil count.

Study design (S): RCTs.

### 2.2 Exclusion criteria

Studies were excluded if they met any of the following conditions: (1) *in vitro* assays or animal experimental research; (2) non-randomized, quasi-randomized or observational clinical designs; (3) combined microbial interventions with confounding adjuvants including vitamin D supplementation and acupuncture; (4) study population comprising participants younger than 18 years; (5) unavailable full-text manuscripts or missing extractable quantitative outcome data.

### 2.3 Search strategy

PubMed, Embase, Cochrane Library, Web of Science, and China National Knowledge Infrastructure (CNKI) were systematically searched from inception to May 2026. The reference lists of included studies were also searched. Search terms in English and Chinese included: asthma, probiotic, prebiotic, synbiotic, *Lactobacillus*, *Bifidobacterium*, *Saccharomyces*, *inulin*, *galacto-oligosaccharide*, randomized controlled trial, and their Chinese equivalents. The detailed search strategy is provided in **Supplementary Table 2**.

### 2.4 Study selection and data extraction

Two reviewers (Qianqian Zhang, Ling Liu) independently screened titles, abstracts, and full texts. Disagreements were resolved by discussion or by consulting a third reviewer (Nianzhi Zhang). A standardized form was used to extract the following data: first author, publication year, country, sample size (intervention/control), patient characteristics (age, sex, asthma severity, background treatment), intervention (strain/component, dose, duration), control measures, and outcome data (mean ± SD post-intervention). For data reported as median and interquartile range (IQR), the method of Wan et al. (15) was used to estimate the mean and SD. For outcomes reported only as P values or in figures, the authors were contacted for raw data; if not obtainable, the outcome was described qualitatively.

### 2.5 Risk of bias assessment

Two reviewers (Qianqian Zhang, Ling Liu) independently assessed the risk of bias using the Cochrane RoB 2.0 tool, covering the following domains: randomization process, deviations from intended interventions, missing outcome data, measurement of the outcome, and selection of the reported result. Each study was judged as being at “low risk of bias,” “some concerns,” or “high risk of bias.” Disagreements were resolved by discussion or by consulting a third reviewer (Nianzhi Zhang).

### 2.6 Grading of evidence quality

The GRADE (Grading of Recommendations, Assessment, Development and Evaluations) system was used to grade the quality of evidence for the primary outcomes, classified as high, moderate, low, or very low.

### 2.7 Pooled meta-analysis

All quantitative pooled syntheses were performed using RevMan 5.4 and Stata 18.0 software. Standardized mean difference (SMD) with corresponding 95% confidence intervals (CIs) was selected as the pooled effect size for continuous outcomes. Considering the anticipated clinical and methodological heterogeneity across trials, the DerSimonian–Laird random-effects model was adopted for all meta-analyses. Heterogeneity was quantified via the I² statistic, with thresholds defined as low (I² < 25%), moderate (25% ≤ I² ≤ 50%) and substantial (I² > 50%). Prespecified subgroup analyses were conducted to explore potential sources of heterogeneity when I² > 50%, stratified by intervention category and treatment duration.

### 2.8 Subgroup, sensitivity analysis and publication bias assessment

Funnel plots and Egger’s regression test were not implemented for publication bias evaluation, as fewer than 10 trials were available for each pooled endpoint; such tests lack sufficient statistical power under limited sample sizes. To mitigate this risk, a comprehensive search of both English and Chinese databases was conducted, and all trials, including those with neutral or non-significant results, were included. **Supplementary Table 3** provides a transparent summary of all positive, neutral, and negative trials.

A prespecified sensitivity analysis was carried out to verify the robustness of primary pooled effects against baseline imbalance. Only RCTs with non-significant between-group differences in baseline FEV_1_ and ACT scores (P > 0.05) were re-analyzed with the identical random-effects model as the main analysis. Pooled effect sizes, heterogeneity indicators and comparative results of the restricted sensitivity dataset are presented in **Supplementary Table 4**.

## 3 Results

### 3.1 Study selection

Initial database searching yielded 1,856 records. After duplicate removal, 712 unique articles underwent title and abstract screening; 631 irrelevant publications were excluded, leaving 81 manuscripts for full-text eligibility assessment. After strict full-text assessment, 68 studies were excluded for failing to satisfy the predefined PICOS criteria, and 13 RCTs were ultimately incorporated into the present review (9, 16–27).

**Figure 1:**
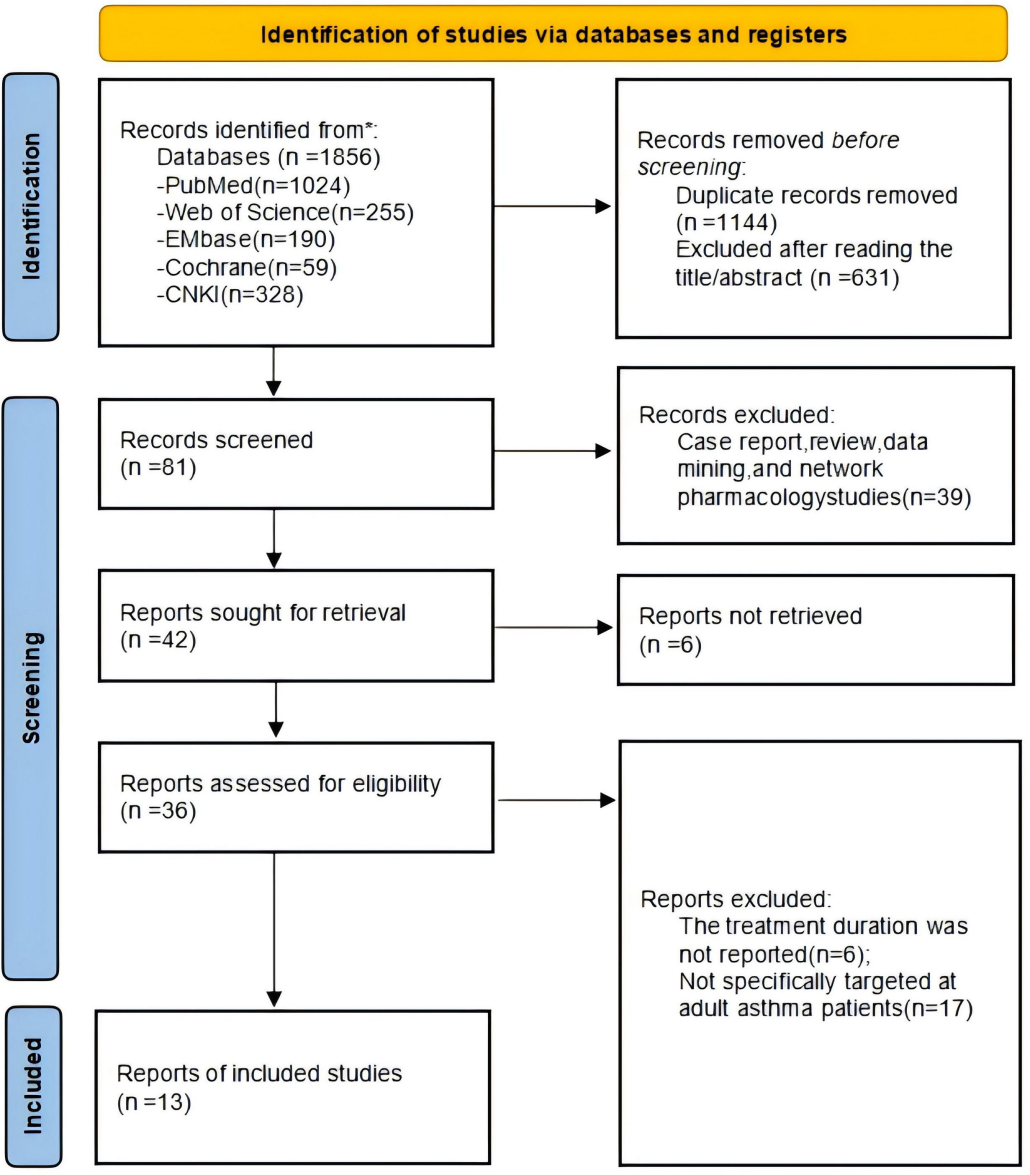
PRISMA flow diagram.

Notably, six of the 13 included trials could not be integrated into quantitative meta-analysis due to incomplete extractable continuous outcome data. Key barriers to quantitative pooling included outcome data reported solely as medians and interquartile ranges without extractable mean and standard deviation values, outcome information presented solely via figures without raw numerical data, and missing complete post-intervention metrics for pulmonary function and inflammatory biomarkers. The remaining seven trials furnished full post-intervention mean ± SD datasets that met the criteria for random-effects pooled synthesis. The full PRISMA study selection flow chart is presented in **Figure 1**.

### 3.2 Characteristics of included studies

The 13 studies (9, 16–27) were published between 2011 and 2025 and conducted in the Netherlands, the United Kingdom, Canada, China, Australia, Iran, and other countries. All studies included adult (≥18 years) asthma patients, with disease severity ranging from mild intermittent to moderate persistent. Interventions included: single probiotics (L. reuteri DSM-17938, L. reuteri CCFM1040, S. boulardii, B. lactis Probio-M8, triple viable Bifidobacterium), prebiotics (B-GOS, inulin), and synbiotics (probiotics + prebiotics/probiotics + AIT). Controls were placebo or usual care. Intervention duration ranged from a single postprandial dose to six months. Detailed characteristics are presented in **Table 1**.

**Table 1:** Basic characteristics of included studies.

First author (year)	Country	Intervention type	Sample size (T/C)	Age (T, years)	Age (C, years)	Asthma stage	Intervention	Control	Duration
van de Pol 2011	Netherlands	Synbiotic	14/15	28.0 (21-51)*	25.0 (18-51)*	Chronic persistent	Synbiotic (B. breve M-16V + scGOS/lcFOS)	Placebo	4 weeks
Williams 2016	UK	Prebiotic	10/10	27±7	27±7	Chronic persistent	Prebiotic B-GOS 5.5 g/d	Placebo	3 weeks
Satia 2021	Canada	Probiotic	15/15	27.3±12.6	27.3±12.6	Chronic persistent	Probiotic L. reuteri DSM-17938	Placebo	4 weeks
Li 2022	China	Probiotic	4/4	41.0±10.6	48.5±6.8	Chronic persistent	Probiotic L. reuteri CCFM1040	Placebo	8 weeks
Liu J 2016	China	Probiotic	14/15	19.8±4.5	23.5±5.6	Chronic persistent	AIT + Clostridium butyricum	AIT + placebo	6 months
Halnes 2017	Australia	Synbiotic	12/17	42.1±3.4	40.4±4.6	Chronic persistent	Single meal: inulin 3.5 g + probiotic yogurt	Mashed potato	Single postprandial
McLoughlin 2019	Australia	Prebiotic	17/17	43 (19-82)*	43 (19-82)*	Chronic persistent	Inulin 12 g/d	Placebo	7 days
Sadrifar 2023	Iran	Synbiotic	17/18	38.6±9.1	38.6±11.9	Chronic persistent	7-strain probiotic + FOS	Placebo	8 weeks
Liu A 2021	China	Probiotic	29/26	–	–	Chronic persistent	B. lactis Probio-M8	Placebo	3 months
Dezfouli 2025	Iran	Probiotic	25/25	42.5±10.6	35.9±14.5	Chronic persistent	S. boulardii 250 mg/d	Placebo	3 months
Li JQ 2025	China	Probiotic	38/38	37.7±4.2	37.6±4.1	Chronic persistent	Bifidobacterium triple viable	Placebo	4 weeks
Lin S 2021	China	Probiotic	103/103	49.7±12.8	50.2±11.6	Acute exacerbation	Bifidobacterium triple viable	Conventional treatment	8 weeks
Sun Q 2025	China	Probiotic	34/34	39.8±5.4*	40.2±5.1*	Chronic persistent	Bifidobacterium triple viable + salmeterol/fluticasone	Salmeterol/fluticasone	4 weeks

Values are presented as mean ± SD or median (range) as indicated; * median (range) or estimated value. T: intervention group; C: control group.

AIT, allergen immunotherapy; B-GOS, galacto-oligosaccharide mixture; FOS, fructo-oligosaccharides; scGOS/lcFOS, short-chain galacto-oligosaccharides/long-chain fructo-oligosaccharides.

### 3.3 Risk of bias assessment

**Figure 2:**
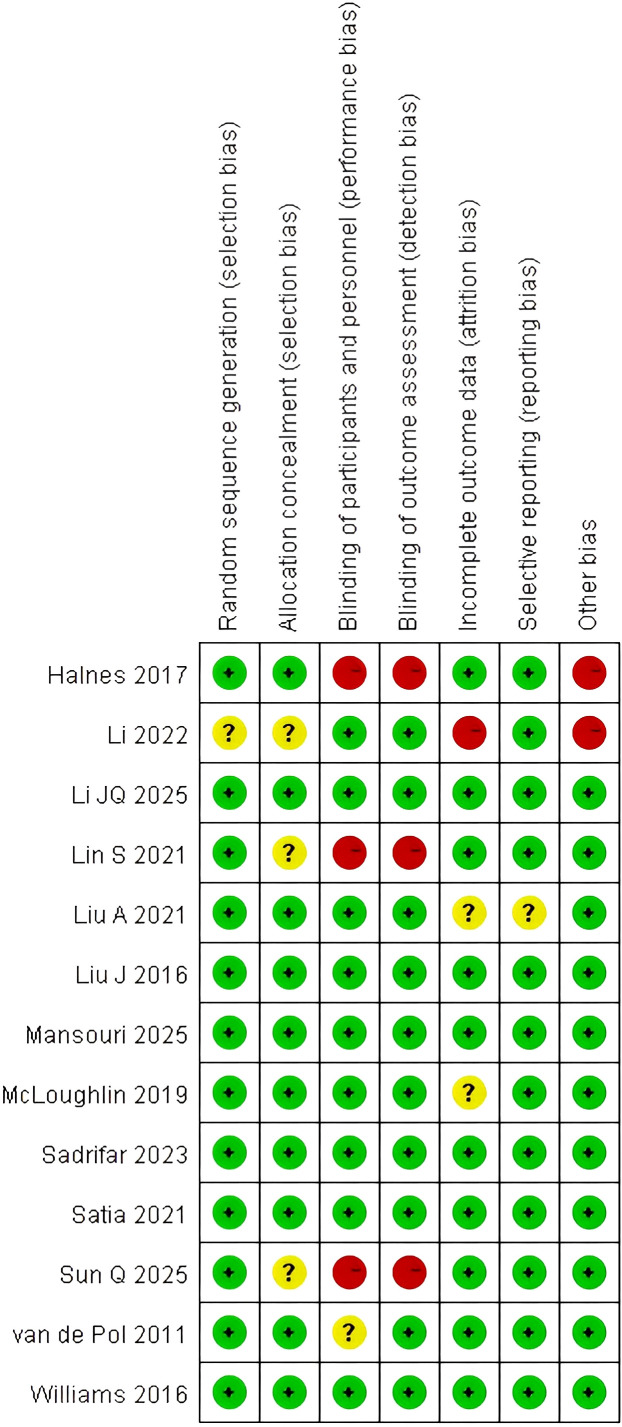
Risk of bias summary.

Risk of bias was assessed using the RoB 2.0 tool for the 13 studies. A summary is presented in **Figure 2**. Results showed: six studies had low risk of bias (9, 17–19, 21, 25); five studies had some concerns (16, 22, 24, 26, 27), mainly due to incomplete outcome data reporting or lack of blinding with placebo; two studies (20, 23) were judged as high risk of bias, mainly due to very small sample size or very short treatment duration.

### 3.4 Quantitative meta-analysis results

#### 3.4.1 Lung function: FEV_1_ (L)

**Figure 3:**
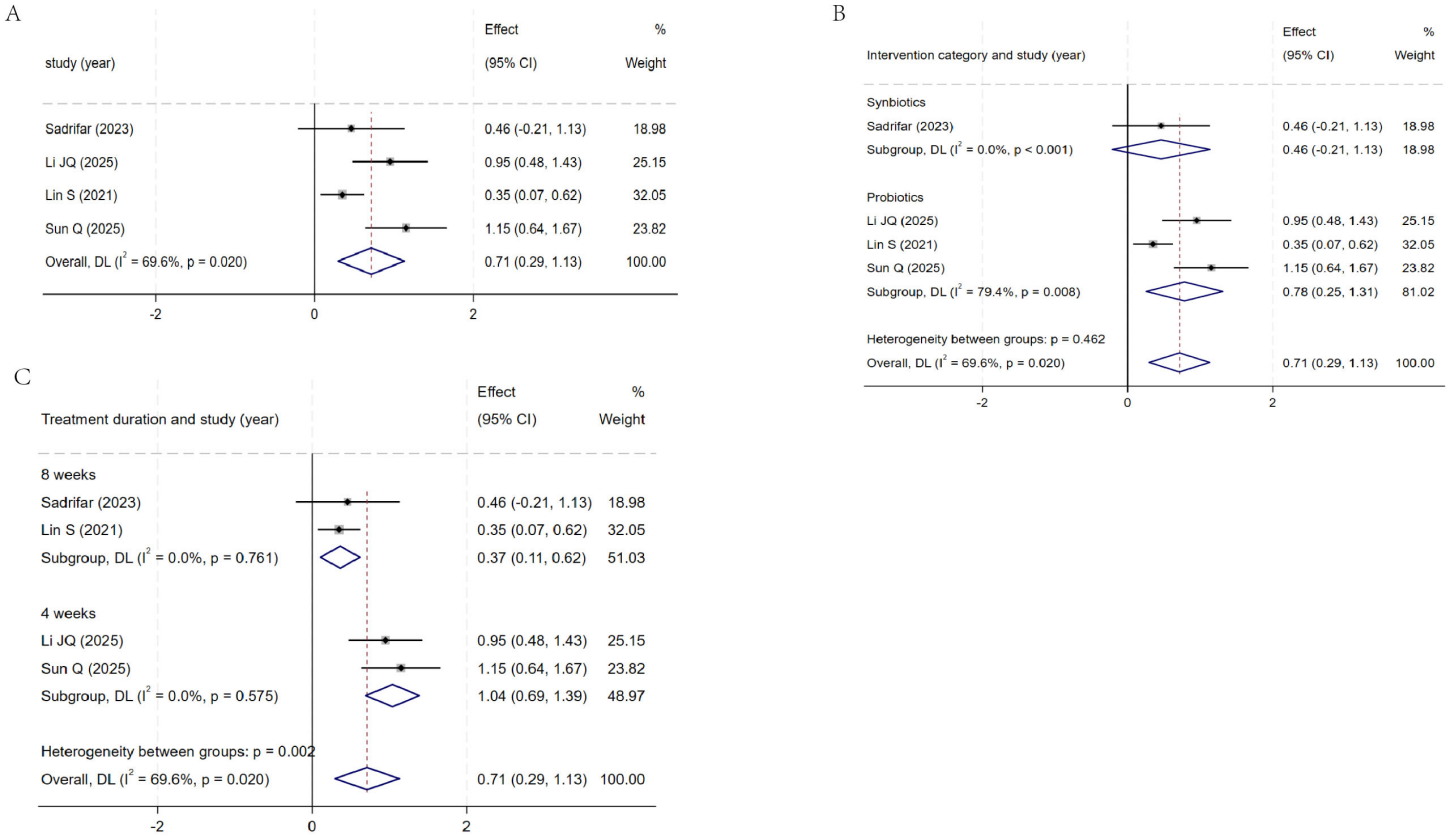
**(A)** Forest plot of FEV_1_; **(B)** Subgroup analysis of FEV_1_ stratified by intervention category; **(C)** Subgroup analysis of FEV_1_ stratified by treatment duration. Weights and between-subgropus heterogeneity test are from random-effects model.

Four studies (9, 25–27) (**Figure 3**) reported complete post-intervention FEV_1_ data. Random-effects pooling yielded significant improvement (SMD=0.71, 95%CI 0.29–1.13, P=0.020) with substantial heterogeneity (I²=69.6%, P=0.020; **Figure 3A**). Two pre-specified subgroup analyses were conducted to explore heterogeneity (**Figure 3B**: intervention type; **Figure 3C**: treatment duration).

Stratified by intervention category: Synbiotics showed non-significant effects (SMD=0.46, 95%CI −0.21–1.13, I²=0.0%), while probiotics significantly elevated FEV_1_ (SMD=0.78, 95%CI 0.25–1.31, P=0.008, I²=79.46%). No significant between-subgroup difference was detected (P=0.462).

Stratified by treatment duration: The 4-week group had a numerically larger effect size (SMD=1.04, 95%CI 0.69–1.39, I²=0.0%) than the 8-week group (SMD=0.37, 95%CI 0.11–0.62, I²=0.0%), with a significant inter-group discrepancy (P=0.002).

#### 3.4.2 Lung function: FEV_1%_ predicted

**Figure 4:**
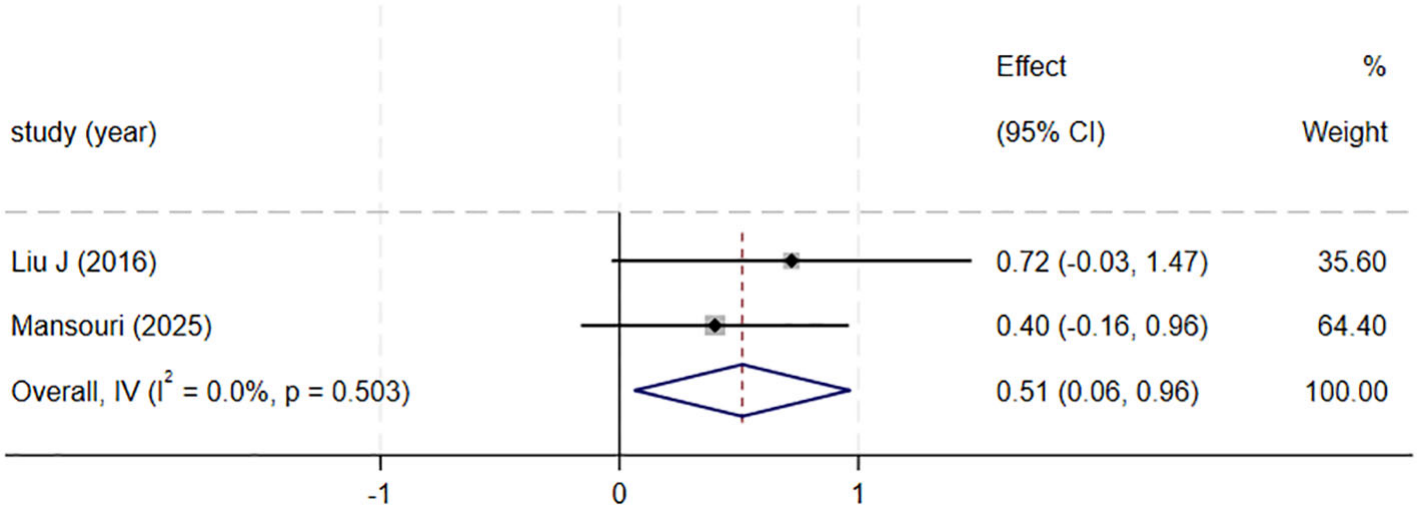
Forest plot for FEV_1%_ predicted.

Two studies (17, 21) reported FEV_1%_ predicted. Heterogeneity between the two studies was low (I²=0%), so a fixed-effect model was used. The pooled result showed that probiotics/synbiotics improved FEV_1%_ predicted (SMD=0.51, 95% CI 0.06-0.96, P=0.026) (**Figure 4**).

#### 3.4.3 ACT score

**Figure 5:**
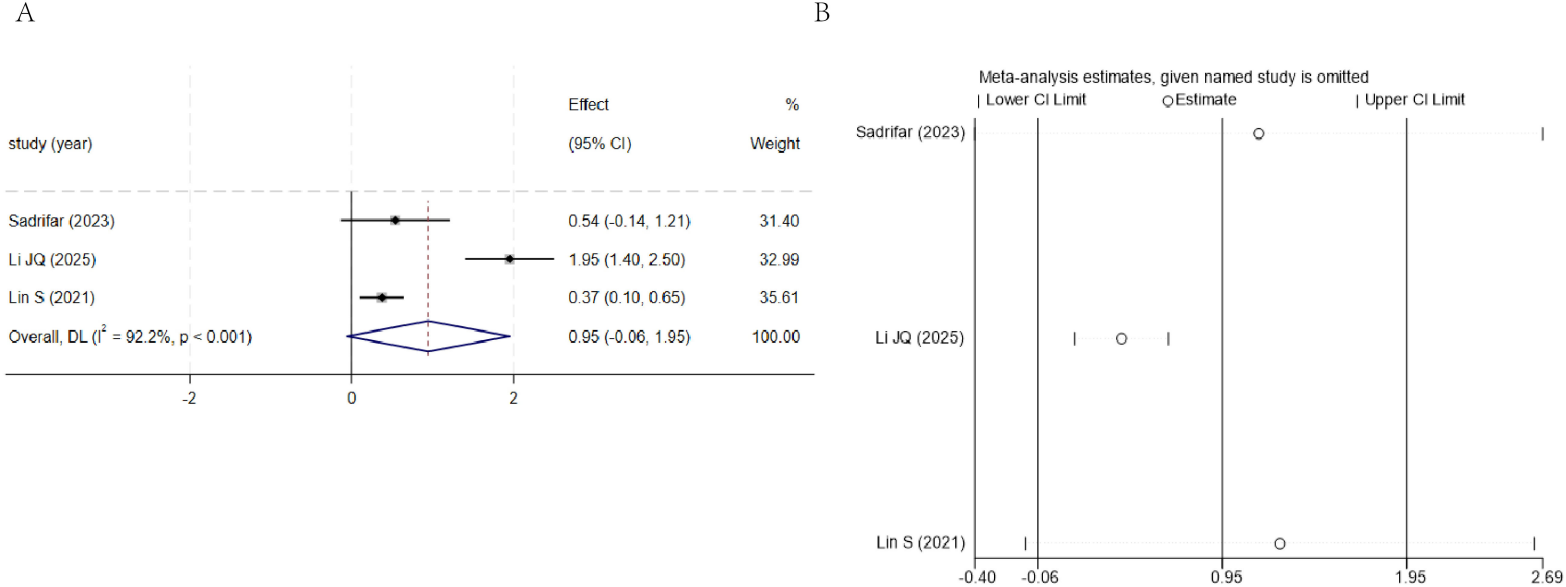
**(A)** Forest plot of ACT scores **(B)** Sensitivity analysis of ACT scores.

Three studies (9, 25, 26) reported ACT scores. Overall heterogeneity was high (I²=92.2%, P<0.001). The random-effects model gave SMD=0.95 (95% CI -0.06 to 1.95, P=0.065). Sensitivity analysis after removing one study (25) gave SMD=0.45 (95% CI 0.21-0.69, I²=0%, P<0.001). This indicates that the overall non-significant effect on the ACT score was highly sensitive to a single study, reflecting inconsistency in the findings (**Figure 5**).

#### 3.4.4 FeNO

Only one study (21) (n=14 in the intervention group, n=15 in the control group) reported FeNO (ppb). The synbiotics + AIT group had 14.8 ± 9.9, and the AIT + placebo group had 24.9 ± 12.9, yielding SMD= -0.88 (95% CI -1.65 to -0.11, P=0.026).

#### 3.4.5 Lung function: FVC

Two studies (17, 27) reported FVC in different units. Kavosh et al. (17) reported FVC % predicted: intervention group 97.20 ± 15.10, control group 95.76 ± 15.33, SMD=0.09 (95% CI -0.46 to 0.64, P=0.74), not significant. Sun Q et al. (27) reported absolute FVC (L): intervention group 1.77 ± 0.85, control group 1.36 ± 0.17, SMD=0.66 (95% CI 0.29-1.03, P=0.001), indicating that probiotics plus usual care improved FVC. Because of different units and only two studies, no meta-analysis was performed.

#### 3.4.6 Inflammatory markers (IL-5, IgE)

Only Dezfouli et al. (17) (n=25/25) reported serum IL-5 and IgE. IL-5: intervention group 72.84 ± 16.81 pg/mL, control group 78.45 ± 12.05 pg/mL, SMD= -0.38 (95% CI -0.94 to 0.18, P=0.18). IgE: intervention group 254.96 ± 381.97 IU/mL, control group 303.36 ± 300.17 IU/mL, SMD= -0.14 (95% CI -0.70 to 0.42, P=0.62). Neither was statistically significant, suggesting that S. boulardii had no significant effect on these two inflammatory markers.

### 3.5 Descriptive summary

PEF-related outcomes were reported in different units across four studies and are therefore presented individually (**Table 2**). Each study demonstrated an enhancement in PEF levels following the respective intervention.

**Table 2:** Effects on PEF or PEF-related indicators.

Study	Outcome definition	Intervention group (mean± SD)	Control group (mean± SD)	MD (95% CI)	P value
Lin S 2021	PEF (L/s)	2.99±0.37 (n=103)	2.81±0.45 (n=103)	0.18 (0.07, 0.29)	0.002
Sun Q 2025	PEF (L/min)	190.34±8.49 (n=34)	170.34±6.94 (n=34)	20.0 (16.3, 23.7)	<0.001
Liu J 2016	PEFR % predicted	98±18 (n=14)	78±15 (n=15)	20.0 (7.4, 32.6)	0.003
Williams 2016	Maximum post-exercise fall in FEV_1_ (mL)	570±310 (n=10)	840±430 (n=10)	-270 (-471, -69)	0.008

### 3.6 Safety

All 13 studies reported that the interventions were generally well tolerated. Mild adverse gastrointestinal reactions were documented in a small number of trials. One trial (16) reported mild bloating, diarrhea or constipation in 3/14 in the probiotic group vs. 7/15 in the placebo group; one study (19) reported one case of upper respiratory tract infection and mild nausea in the probiotic group. No other studies reported significant adverse reactions.

### 3.7 GRADE evidence quality

The GRADE framework was applied to assess evidence certainty for all primary endpoints, with ratings ranging from low to moderate due to risks of bias, imprecision and potential publication bias (**Table 3**).

**Table 3:** GRADE evidence profile.

Outcome	Number of studies	Risk of bias	Inconsistency	Indirectness	Imprecision	Publication bias	Quality of evidence
FEV_1_ (L)	4	Not serious	Moderate	Not serious	Not serious	Suspected	⊕⊕⊕○ Moderate
FEV_1_ % predicted	2	Not serious	None	Not serious	Serious	Suspected	⊕⊕○○ Low
ACT score	3	Not serious	Serious	Not serious	Not serious	Suspected	⊕⊕○○ Low
PEF (individual studies)	4	Not serious	Serious	Not serious	Not serious	Suspected	⊕⊕⊕○ Moderate
FeNO	1	Not serious	None	Not serious	Serious	Suspected	⊕⊕○○ Low

Absolute FEV_1_ received moderate-certainty evidence; one level was downgraded for partial baseline imbalance across trials, while consistent beneficial pooled effects sustained moderate confidence.

Evidence for ACT scores was graded low, mainly attributable to substantial heterogeneity in intervention strains and treatment durations.

FeNO was rated very low certainty, as extractable data were limited to a single small RCT with no replicable multi-trial pooled evidence.

## 4 Discussion

This systematic review incorporated 13 RCTs, among which 7 provided eligible continuous quantitative data for meta-analytical synthesis. The effects of probiotics, prebiotics, and synbiotics on lung function, asthma symptom control, and airway inflammatory markers in adult asthmatic patients were systematically assessed.

### 4.1 Regulatory mechanisms and clinical effects of probiotics, prebiotics and synbiotics in adult asthma

This meta-analysis suggests that all three types of gut microecological interventions possess potential regulatory effects, with therapeutic efficacy appearing to be influenced by strain combination and formulation type. Multi-strain compound preparations of Lactobacillus and Bifidobacterium synergistically reshape systemic immune homeostasis via multiple gut-lung axis pathways (28). On one hand, they directly suppress Th2 pro-inflammatory cytokines such as IL-4 and IL-13, upregulate IFN-γ, and induce Treg cells to secrete IL-10 and TGF-β, thereby alleviating airway hyperresponsiveness (29). On the other hand, they ferment to produce SCFAs including acetate and propionate, which reduce eosinophilic airway infiltration through histone deacetylase inhibition. Meanwhile, these bacteria upregulate tight junction proteins to repair the intestinal epithelial barrier and block the inflammatory cascade in the lung triggered by translocation of allergens and endotoxins (30). Tryptophan metabolites derived from gut microbiota further activate the AhR signaling pathway in alveolar macrophages to persistently inhibit the release of TNF-α and IL-6 (31).

The number of eligible single-strain RCTs was limited, with substantial inter-trial heterogeneity in viable bacterial dosage and participants’ baseline intestinal microecology. Compared with multi-strain blends, single strains generate a narrower panel of anti-inflammatory metabolites and are less capable of synchronously activating multiple gut-lung axis immune cascades. Combined with the above confounding factors, these features contributed to inconsistent lung function and inflammatory biomarker outcomes across trials, with no consistent positive therapeutic signals observed. The higher effect size observed in the 4-week short-term intervention group was an exploratory statistical observation, and no reliable molecular or clinical data currently explain the efficacy differences associated with intervention duration. Notably, none of the included human trials concurrently detected gut-lung axis mediators such as fecal microbiota, circulating SCFAs and peripheral T cell subsets. Therefore, the complete regulatory pathways observed in animal models lack direct human clinical verification.

Synbiotics utilize prebiotics such as fructooligosaccharides (FOS) and galactooligosaccharides (GOS) to enhance intestinal colonization of probiotics, theoretically conferring synergistic anti-inflammatory effects (32). However, clinical evidence to date remains inconsistent. Small-scale trials indicated that synbiotics combined with allergen-specific immunotherapy slightly reduced FeNO, yet no consistent positive changes were observed in systemic inflammatory markers such as IL-5 and IgE. The pooled effect of ACT scores showed no statistical significance, and only patients with extremely poor baseline asthma control exhibited mild symptomatic relief. Mechanistically, controversies remain: prebiotics independently enrich beneficial commensals and boost SCFA production. Nevertheless, a three-arm randomized double-blind crossover trial demonstrated that inulin supplementation alone significantly improved airway inflammation and asthma control indicators, whereas combined inulin-probiotic intervention yielded no additional benefits (24). Large clinical cohorts and molecular mechanistic investigations are still required to verify whether synergistic additive effects exist between probiotics and prebiotics.

Standalone prebiotics directly act as intestinal fermentation substrates to remodel microecology and mitigate airway hyperresponsiveness (33), yet relevant clinical trials in adults are scarce. Limited existing studies confirmed that GOS and inulin alleviate hyperventilation-induced bronchoconstriction and ameliorate airway inflammation. Few trials within our included literature established isolated prebiotic intervention arms, and long-term large-cohort data are lacking to validate their independent regulatory pathways and sustained clinical benefits.

### 4.2 Distinctive innovations of the present review relative to previous systematic reviews

Most existing meta-analyses of microecological interventions for asthma either exclusively recruited pediatric patients or pooled mixed adult and pediatric data for combined synthesis. Most of these studies only evaluated probiotics alone, lacking quantitative head-to-head comparisons across prebiotics and synbiotics (13, 14, 34). A detailed comparative summary of the present analysis versus prior reviews is displayed in **Supplementary Table 5**. Compared with previous work, this study presents three core innovations. First, participants were strictly restricted to adults aged ≥18 years, with pediatric and adolescent data excluded. This design eliminated confounding biases derived from age-dependent immune characteristics and filled the evidence gap of a dedicated meta-analysis integrating three microecological interventions specifically for adult asthma. Second, direct and indirect comparisons of the three interventions were performed within a unified statistical framework, clearly distinguishing efficacy patterns across different strains and formulations. Third, sensitivity analyses were conducted on subsets balanced for baseline lung function and ACT scores to adjust for confounding variables. All core outcomes were graded for evidence quality in strict accordance with the GRADE framework, markedly improving the differentiation and rigor of evidence-based conclusions.

### 4.3 Heterogeneity and overall limitations of the study

Significant between-study heterogeneity was identified alongside multiple methodological limitations. Primary sources of heterogeneity were as follows:

Variations in intervention formulations: single-strain probiotics, multi-strain blends, pure prebiotics and synbiotics with disparate ratios modulate distinct metabolic pathways, constituting the dominant source of FEV1 heterogeneity.

Mixed intervention durations: trials with 4-week and 8-week regimens coexisted, accompanied by disparities in participants’ baseline lung function and baseline inhaled corticosteroid regimens.

Non-standardized outcome reporting: inconsistent units for PEF and FVC, and numerous studies only reporting interquartile ranges rather than continuous data eligible for pooling. Heterogeneity in ACT scores stemmed from large disparities in baseline asthma control across cohorts.

Uneven geographical and disease severity distribution of participants: included studies predominantly recruited Asian and European populations, with insufficient samples of patients with severe asthma.

Intrinsic limitations of this meta-analysis are summarized below. Each pooled outcome was supported by fewer than 10 trials, rendering funnel plots and Egger’s regression unsuitable for quantitative publication bias evaluation. Six out of 13 RCTs lacked extractable continuous quantitative data, which reduced the statistical power of the quantitative syntheses. Confounding variables including strain type, viable dosage and baseline maintenance therapy could not be fully disentangled. No trials measured gut-lung axis-related biomarkers, resulting in a lack of human-derived mechanistic evidence. The maximum follow-up duration across all trials was only six months, with no long-term data regarding sustained efficacy and safety. Multiple Chinese and English databases were comprehensively searched, and all published positive, negative and neutral studies were included. Nevertheless, publication bias originating from unpublished negative trials cannot be fully ruled out.

### 4.4 Implications for clinical practice

Evidence from this meta-analysis suggests gut microecological preparations should only serve as individualized adjuvant therapies for adult asthma and are not recommended for routine use in all patients. Multi-strain compound formulations containing *Bifidobacterium* and *Lactobacillus* possess potential benefits for lung function improvement, while anti-inflammatory and symptom-controlling evidence for single-strain probiotics and synbiotics remains unstable. Clinicians may consider factors such as baseline asthma control and habitual dietary fiber intake when assessing the potential for a therapeutic response. For adults with frequent wheezing, recurrent respiratory infections and suboptimal control under standard inhaled medication, compound microecological preparations may be administered as adjunctive treatment. Clinicians must fully inform patients of the limitations of current evidence and implement shared decision-making throughout treatment. Gastrointestinal tolerance should be regularly monitored throughout supplementation, and preparations should be discontinued if persistent bloating or diarrhea occurs. All microecological supplements cannot replace first-line foundational asthma medications including inhaled glucocorticoids and long-acting β_2_ agonists.

### 4.5 Directions for future clinical trials

Standardized randomized controlled trials are warranted to clarify optimal strain combinations, effective viable bacterial dosages and appropriate intervention durations, and to determine whether synergistic benefits exist when probiotics and prebiotics are co-administered. Uniform reporting standards for lung function, ACT and inflammatory biomarkers should be established, with simultaneous detection of gut microbiota, SCFAs and peripheral immune markers to complete the human gut-lung axis regulatory evidence chain. Longitudinal trials with at least 12 months of follow-up are required to assess sustained efficacy and safety using hard clinical endpoints including asthma exacerbations and emergency department visits. Multi-omics technologies can be applied to dissect the complete molecular pathways by which microecological interventions regulate airway inflammation, facilitating the construction of a standardized evidence-based system for gut-targeted adjuvant therapy in adult asthma.

## 5 Conclusion

This meta-analysis provides moderate-quality evidence that multi-strain probiotics or synbiotics containing Bifidobacterium and Lactobacillus may yield mild improvements in lung function in adults with asthma. However, existing evidence is insufficient to support the routine clinical use of microecological supplements for adult asthma. Large-scale, long-term, rigorously designed randomized controlled trials are required to further validate the beneficial signals observed in this analysis and establish standardized adjuvant treatment protocols.

## Data Availability

The original contributions presented in the study are included in the article/**Supplementary Material**. Further inquiries can be directed to the corresponding authors.
