# Supplementary material for: Effects of probiotics, prebiotics, and synbiotics on lung function and symptoms in adults with bronchial asthma: a systematic review and meta-analysis
Source: Front Immunol. 2026 Aug 4;17:1896757. doi: 10.3389/fimmu.2026.1896757 (PMC13481230; doi:10.3389/fimmu.2026.1896757)
Supplement: Supplementary file 1 [file Table4.docx]

**Identification of studies via databases and registers**

Records removed *before screening*:

Duplicate records removed (n =1144)

Excluded after reading the title/abstract (n =631)

Records identified from*:

Databases (n =1856)

-PubMed(n=1024)

-Web of Science(n=255)

-EMbase(n=190)

-Cochrane(n=59)

-CNKI(n=328)

**Identification**

Records excluded:

Case report,review,data mining,and network pharmacologystudies(n=39)

Records screened

(n =81)

Reports sought for retrieval

(n =42)

Reports not retrieved

(n =6)

**Screening**

Reports assessed for eligibility

(n =36)

Reports excluded:

The treatment duration was not reported(n=6);

Not specifically targeted at adult asthma patients(n=17)

Reports of included studies

(n =13)

**Included**

*Consider, if feasible to do so, reporting the number of records identified from each database or register searched (rather than the total number across all databases/registers).

**If automation tools were used, indicate how many records were excluded by a human and how many were excluded by automation tools.

Source: Page MJ, et al. BMJ 2021;372:n71. doi: 10.1136/bmj.n71.

This work is licensed under CC BY 4.0. To view a copy of this license, visit <https://creativecommons.org/licenses/by/4.0/>
