# Supplementary material for: Effects of probiotics, prebiotics, and synbiotics on lung function and symptoms in adults with bronchial asthma: a systematic review and meta-analysis
Source: Front Immunol. 2026 Aug 4;17:1896757. doi: 10.3389/fimmu.2026.1896757 (PMC13481230; doi:10.3389/fimmu.2026.1896757)
Supplement: Supplementary file 2 [file Table5.docx]

**Effects of probiotics, prebiotics, and synbiotics on pulmonary function and symptoms in adult patients with bronchial asthma: A systematic review and meta-analysis**

**Supplement: content**

| **TABLE S1a. Search strategy in Pubmed** ([https://pubmed. ncbi. nlm. nih. gov/](https://pubmed.ncbi.nlm.nih.gov/)) | |
| --- | --- |
| **Asthma （P）** | 1. Asthma[MeSH Terms]  2. Bronchial Asthma[Title/Abstract]  3. Allergic Asthma[Title/Abstract]  4. 1 OR 2 OR 3 |
| **Probiotics/Prebiotics/Hyphenobiotics（I）** | 5. Probiotics[MeSH Terms]  6. Probiotic[Title/Abstract]  7. Prebiotics[MeSH Terms]  8. Prebiotic[Title/Abstract]  9. Synbiotics[Title/Abstract]  10. Lactobacillus[MeSH Terms]  11. Bifidobacterium[MeSH Terms]  12. Saccharomyces boulardii[Title/Abstract]  13. Inulin[Title/Abstract]  14. Galacto-oligosaccharide[Title/Abstract]  15. Gut Microbiota[MeSH Terms]  16. 5 OR 6 OR 7 OR 8 OR 9 OR 10 OR 11 OR 12 OR 13 OR 14 OR 15 |
| **Study Type** | 17. Randomized Controlled Trial[Publication Type]  18. Clinical Trial[Publication Type]  19. 17 OR 18 |
| **Combine** | 20. 4 AND 16 AND 19 |

| **TABLE S1b. Search strategy in EMBASE (**<https://www.embase.com/>**)** | |
| --- | --- |
| **Asthma （P）** | #1 'asthma'/exp  #2 'bronchial asthma':ti,ab,kw  #3 'allergic asthma':ti,ab,kw  #4 #1 OR #2 OR #3 |
| **Probiotics/Prebiotics/Hyphenobiotics（I）** | #5 'probiotic'/exp  #6 'prebiotic'/exp  #7 'synbiotic'/exp  #8 'lactobacillus'/exp  #9 'bifidobacterium'/exp  #10 'saccharomyces boulardii':ti,ab,kw  #11 'inulin':ti,ab,kw  #12 'galacto-oligosaccharide':ti,ab,kw  #13 'gut microbiota'/exp  #14 #5 OR #6 OR #7 OR #8 OR #9 OR #10 OR #11 OR #12 OR #13 |
| **Study Type** | #15 'randomized controlled trial'/exp  #16 'clinical trial'/exp  #17 #15 OR #16 |
| **Combine** | #18 #4 AND #14 AND #17 |

| **TABLE S1c. Search strategy in Web Of Science(**<https://www.webofscience.com/>**)** | |
| --- | --- |
| **Asthma （P）** | 1. TS=(Asthma)  2. TS=("Bronchial Asthma")  3. TS=("Allergic Asthma")  4. 1 OR 2 OR 3 |
| **Probiotics/Prebiotics/Hyphenobiotics（I）** | 5. TS=(Probiotic OR Probiotics)  6. TS=(Prebiotic OR Prebiotics)  7. TS=(Synbiotic OR Synbiotics)  8. TS=(Lactobacillus)  9. TS=(Bifidobacterium)  10. TS=("Saccharomyces boulardii")  11. TS=(Inulin)  12. TS=("Galacto-oligosaccharide")  13. TS=("Gut Microbiota")  14. 5 OR 6 OR 7 OR 8 OR 9 OR 10 OR 11 OR 12 OR 13 |
| **Study Type** | 15. TS=("Randomized Controlled Trial")  16. TS=("Clinical Trial")  17. 15 OR 16 |
| **Combine** | 18. 4 AND 14 AND 17 |

| **TABLE S1d. Search strategy in Cochrane(**<https://www.cochrane.org/>**)** | |
| --- | --- |
| **Asthma （P）** | #1 MeSH descriptor: [Asthma] explode all trees |
| **Probiotics/Prebiotics/Hyphenobiotics（I）** | #2 (probiotic):ti,ab,kw  #3 (prebiotic):ti,ab,kw  #4 (synbiotic):ti,ab,kw  #5 (lactobacillus):ti,ab,kw  #6 (bifidobacterium):ti,ab,kw  #7 ("saccharomyces boulardii"):ti,ab,kw  #8 (inulin):ti,ab,kw  #9 ("galacto-oligosaccharide"):ti,ab,kw  #10 ("gut microbiota"):ti,ab,kw  #11 #2 OR #3 OR #4 OR #5 OR #6 OR #7 OR #8 OR #9 OR #10 |
| **Study Type** | #12 ("randomized controlled trial"):ti,ab,kw  #13 (RCT):ti,ab,kw  #14 #12 OR #13 |
| **Combine** | #15 #1 AND #11 AND #14 |

| **TABLE S1e. Search strategy in Commonly Used Databases for Research in China** | |
| --- | --- |
| **CNKI (**<https://www.cnki.net/>**)** | SU%=(Probiotics + Lactobacillus + Bifidobacterium + Saccharomyces boulardii + Prebiotics + Galactooligosaccharides + Inulin + Synbiotics + Gut Microflora + Microecological Preparations) AND SU% = (Bronchial Asthma + Asthma + Allergic Asthma) AND SU% = (Randomized Controlled Trials + Randomized + Clinical Studies) |
